# Improving Stroke Treatment Using Magnetic Nanoparticle Sensors to Monitor Brain Thrombus Extraction

**DOI:** 10.3390/s25030672

**Published:** 2025-01-23

**Authors:** Dhrubo Jyoti, Daniel Reeves, Scott Gordon-Wylie, Clifford Eskey, John Weaver

**Affiliations:** 1 LCD Nanotech, Hanover, NH 03755, USA; scott@flowaluminum.com; 2 Thayer School of Engineering, Dartmouth College, Hanover, NH 03755, USA; 3 Fred Hutch Cancer Center, University of Washington, Seattle, WA 98195, USA; dreeves@fredhutch.org; 4 Flow Aluminum, Albuquerque, NM 87123, USA; 5 Department of Radiology, Dartmouth Health, Lebanon, NH 03756, USA

**Keywords:** stroke, magnetic nanoparticles, mechanical thrombectomy, clot, thrombus, magnetic dipole field, simulations

## Abstract

(1) Background: Mechanical thrombectomy (MT) successfully treats ischemic strokes by extracting the thrombus, or clot, using a stent retriever to pull it through the blood vessel. However, clot slippage and/or fragmentation can occur. Real-time feedback to a clinician about attachment between the stent and clot could enable more complete removal. We propose a system whereby antibody-targeted magnetic nanoparticles (NPs) are injected via a microcatheter to coat the clot, oscillating magnetic fields excite the particles, and a small coil attached to the catheter picks up a signal that determines the proximity of the clot to the stent. (2) Methods: We used existing simulation code to model the signal from NPs distributed on a hemispherical clot with three orthogonally applied magnetic fields. An in vitro apparatus was built that applied fields and read out signals from a 1.5 mm pickup coil at a variable distance and orientation angle from a sample of 100 nm iron oxide core/shell NPs. (3) Results: Our simulations suggest that the sum of the voltages induced in the pickup coil from three orthogonal applied fields could localize a clot to within 180 µm, regardless of the exact orientation of the pickup coil, with further precision added via rotation-correction formulae. Our experimental system validated simulations; we estimated an in vitro distance recovery precision of 41 µm with a pickup coil 1 mm from the clot. (4) Conclusions: Magnetic NP sensing could be a safe and real-time method to estimate whether a clot is attached to the stent retriever during MT.

## 1. Introduction

There are about 800,000 strokes in the US every year, causing 1 in every 20 deaths and even more frequently resulting in temporary or permanent disability [1]. Nine out of ten strokes are ischemic; a thromboembolism occludes or narrows a blood vessel in the neck or head, resulting in insufficient blood flow and nutrient delivery to part of the brain. Many of the most severe ischemic strokes result from the occlusion of large- or medium-sized arteries, termed large vessel occlusion (LVO) or medium vessel occlusion (MeVO), respectively. Medical therapy with thrombolytic medications is often ineffective for this form of stroke.

However, mechanical thrombectomy (MT)—removal of the thromboembolism via intra-arterial catheters—can produce rapid restoration of blood flow and minimize the area of irreversible ischemic brain injury. Large multicenter randomized controlled trials have demonstrated that MT markedly reduces the severity of brain damage and improves patient outcomes [2] if performed 8 h [3] or even 24 h after the onset of symptoms [4]. MT has revolutionized the treatment of stroke [2,5,6].

Two methods of MT are common. One uses aspiration via a large-bore catheter while the other uses a stent retriever (SR) to pull a thrombus into the catheter. A SR is a metal mesh that expands into the thrombus, enabling the SR to extract the thrombus as it is pulled back into the catheter. The type of thrombus and vessel anatomy can determine the better method, and a serial or simultaneous combination of the two is commonly employed. The process of MT is performed percutaneously, and it is guided by fluoroscopy. The injection of radiopaque contrast opacifies the affected artery up to the point of occlusion and permits the minimally traumatic introduction of the devices used to remove the thrombus. However, this imaging does not directly demonstrate the clot itself, nor does it allow one to see how effectively the clot has been removed during the periods of active device deployment when the thrombus is being removed. During these periods, the thrombus may move partially or not at all, and there is a possibility of thrombus fragmentation. These phenomena can result in failed attempts, and each attempt prolongs the duration of inadequate blood flow. Further, each attempt carries a risk of thrombus fragmentation with the passage of the thrombus to distal locations inaccessible to the retrieval devices. Real-time feedback on thrombus behavior may allow for optimized retrieval techniques. For instance, pulling an SR around a tortuous artery might be optimized by modulating the amount of suction or the speed of extraction.

The MT process is complex and many unknown factors impact clot removal. Methods that characterize the composition and porosity of the clot have been the most successful methods to guide MT, but they can only be performed prior to the procedure, not during the procedure. Numerous publications describe attempts to classify clots prior to the MT procedure using non-contrast CT [7,8] or contrast CT to evaluate the extent that contrast is able to penetrate the clot, termed “perviousness” [9,10]. Imaging metrics have limitations [10,11] but pervious clots are associated with better responses to all therapies, including using drugs like tissue plasminogen activator tPA to dissolve it [12,13]. Clot composition is likely to determine the surface area available to thrombolytic agents, the interaction of the clot with the blood vessel wall [14], and the integration of the SR with the clot. A few studies have suggested that the optimum means of clot extraction may depend on such measures of clot composition [15]. CT and MRI cannot be used to image the thrombus during the procedure because the contrast is too low and efforts to use agents like gold NPs to mark the surface of the clot have had limited success due to low sensitivity [16]. There is no currently available method for measuring clot composition or the integrity of a clot during fluoroscopically guided MT. There is a significant nascent but very promising effort to apply magnetic particle imaging (MPI) to intravascular applications [17,18,19,20], including the evaluation of cerebral perfusion and stroke [21,22], but no method of tracking clots has emerged.

Our goal is to provide a novel sensing tool that will enable physicians to completely remove large vessel thromboembolism rapidly and on the first pass. Direct feedback of thrombus slippage and fragmentation will provide the physician with the information needed to modulate the force they exerted on the SR to maximize efficient and complete clot extraction.

**Figure 1:**
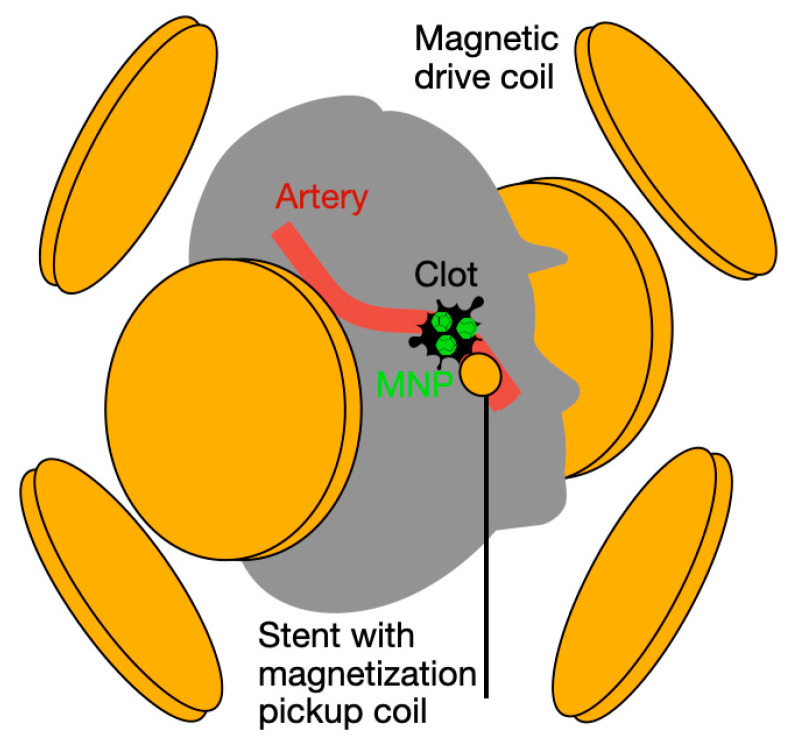
Schematic of novel sensing modality. Three orthogonal pairs of driving magnetic field coils (yellow), centered approximately around the thrombus. The thrombus (clot) is coated by injected targeted NPs. The resulting NP magnetization signal is picked up by a fine-gauge magnetization pickup coil attached to the stent and inserted into the artery (yellow). Thrombus breakage or slippage is detectable via a drop in the pickup coil signal. The change in signal is high with any movement of the thrombus relative to the pickup coil because the signal drops as one over the distance cubed.

We are developing a method for monitoring the location of a thrombus relative to the SR or the aspiration catheter. The signal in coils integrated into the SR or catheter induced by NPs bound to the thrombus is very sensitive to the intervening distance, enabling a sensitive measure of slippage and fragmentation. This method has the potential to provide real-time feedback on clot location and integrity during MT (Figure 1), allowing a physician to adapt the force used and the speed of extraction to minimize fragmentation and associated embolic insult. Our technology will assist clinicians in finding the optimum speed of clot extraction in order to extract the intact clot in the first pass and confirm full extraction. We demonstrate the method and evaluate the expected spatial resolution and sensitivity using simulations. We then confirm the simulation results using experimental in vitro studies. We demonstrate the potential to reliably detect in vivo thrombus slippage or fragmentation during the MT procedure.

## 2. Materials and Methods

### 2.1. Conceptual System: NP-Coated Thrombus

**Figure 2:**
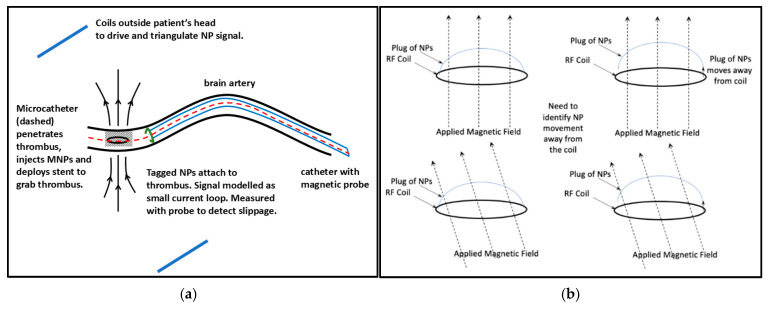
Schematics of (**a**) NP injection and thrombus coating via microcatheter and (**b**) thrombus slippage with applied field orientation. The fine-gauge probe (pickup coil) (green) is near the thrombus (shaded). In (**a**), only one pair out of the three pairs of applied field coils in the full setup is shown.

We modeled a thrombus inside a brain artery as a sphere of diameter 1 mm, equal to the approximate internal diameter of an artery. A stent is inserted through an artery in the leg or arm and passed through the body until it reaches the brain artery and penetrates the thrombus (Figure 2). A micro-catheter is used to inject the NPs on the “far side” of the thrombus. Some NPs may wash off while others bind to the thrombus via antibody targeting [23]. Most stent-retrievers are non-magnetic nickel–titanium alloy, so there is no magnetic excitation signal noise.

### 2.2. Simulation Assumptions

**Figure 3:**
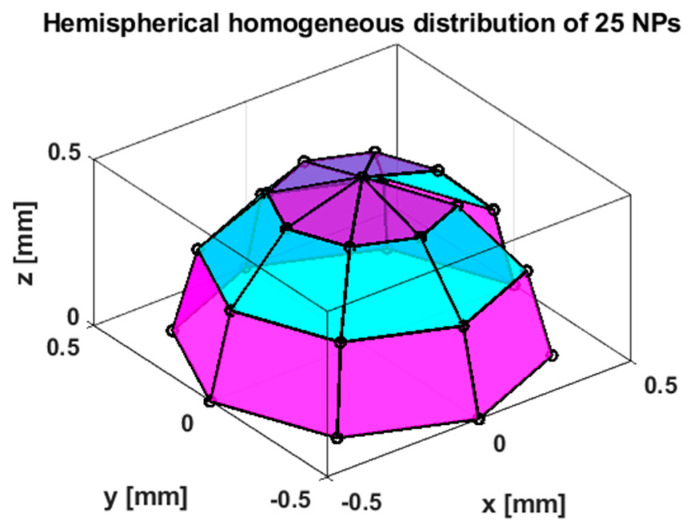
Model assumption of 25 NPs symmetrically distributed on half of a spherical thrombus with a 1 mm diameter. Vertices (dots) indicate the locations of Amperian loops that simulate the NP magnetic field.

We modeled the NPs as having reached a steady state coating the thrombus, i.e., distributed homogeneously on a hemispherical surface (Figure 3). We modeled the magnetic fields emanating from the NPs as those from small Ampèrian loops with a diameter of 0.2 mm. We evenly distributed 25 of these small loops on the hemisphere (Figure 3), a large enough number to cover the surface.

The total magnetic field is the sum of the Amperian loop contributions

(1) $${\overset{⃑}{B}}_{thrombus}(\overset{⃑}{r},t)=\sum_{i=1}^{25}{\overset{⃑}{B}}_{Amperian,\;i}(\overset{⃑}{r},t)$$

The Amperian loop magnetic field ${\overset{⃑}{B}}_{Amperian}$ has separable spatial and time dependence,

(2) $${\overset{⃑}{B}}_{Amperian}^{j}\left(\overset{⃑}{r},\;t\right)={\;M}_{NP}^{j}\left(t\right)\;{\overset{⃑}{B}}_{Amperian}^{j}\left(\overset{⃑}{r}\right)\;$$

where the index j refers to x, y, or z-component. $M_{NP}^{j}\left(t\right)$ is the magnetization as a function of time for a given NP specification and external driving fields. We calculated the NP magnetization below using a Brownian motion simulation that our group previously published [24,25,26]. The spatial dependence $B_{Amperian}^{j}\left(\overset{⃑}{r}\right)$ has the following analytical formula for the special on-axis case:

(3) $${\overset{⃑}{B}}_{Amperian}^{j}\left(z_{j}\right)=\frac{\mu_{0}IR^{2}}{2{\left({z_{j}}^{2}+R^{2}\right)}^{3/2}}{\hat{z}}_{j}$$

where $\mu_{0}$ is the magnetic permeability constant; I is the amplitude of the current in the loop; R is the radius of the loop, which is 0.1 mm in our case; and finally, $z_{j}$ is the distance to the test point on the jth loop’s axis. For an arbitrary location $\overset{⃑}{r}$ (off-axis), a closed-form solution does not exist, but it is straightforward to compute the field numerically [27,28]. The time-varying total flux Φ from the NPs generates an E.M.F. voltage in the pickup coil in the artery:

(4) $$V_{pickup}\left(t\right)=-N_{turns}\;\frac{d}{dt}\Phi \left(t\right)\;.$$

where $N_{turns}$ is the number of turns (windings) in the pickup coil.

### 2.3. Magnetic Flux Calculation

The magnetic signal from the NPs is measured by a single circular pickup coil of 1 mm diameter on the stent, intended to reach as close to the thrombus as possible for maximum signal. The flux of the magnetic field $\overset{⃑}{B}(\overset{⃑}{r})$ through a pickup coil loop is a scalar number given by the surface integral on the circular surface S enclosed by the pickup coil loop,

(5) $$\Phi =\iint_{S}{\overset{⃑}{B}}_{thrombus}\left(\overset{⃑}{r}\right)\;\cdot \;\overset{⃑}{da}$$

where $\overset{⃑}{da}$ is the normal vector for each small patch of surface area inside the loop, with its norm equal to the area of the patch.

We calculate the magnetic flux through the pickup coil Φ numerically by dividing up the circular surface enclosed by the pickup coil loop into a two-dimensional circular grid with even spacing. Each element *j* of the grid is a little square. The normal vector $\overset{⃑}{da_{j}}$ is perpendicular to the square with a norm equal to the area of the square. The integrand in Equation (5) is calculated for each of these squares and summed to obtain the total flux Φ. That is, the surface integral in Equation (5) was computed as a sum over dot products:

(6) $$\Phi =\sum_{j}\;{\overset{⃑}{B}}_{thrombus}\left({\overset{⃑}{r}}_{j}\right)\;\cdot \;\overset{⃑}{da_{j}}$$

where ${\overset{⃑}{r}}_{j}$ is the distance vector from the thrombus to the little square *j*. We ensured accuracy by calculating over a finer and finer grid until the results were stable. We found a less than 2% change in the total flux when the dimension of the little square was halved from 0.0125 micron (corresponding to about 80,000 times 80,000 grid squares over the 1 mm circular loop) to 0.00625 micron. The MATLAB 2023a code that we developed for the simulation uses numerical elliptical integration for computing off-axis fields [27]. Our code takes only a few minutes to run on a personal computer to calculate the total flux through the pickup coil.

The magnetic field emanating from the NPs is complicated and lacks a simple analytical formula as a function of distance. However, we expect the coil flux from the hemispherical distribution of NPs to have roughly an inverse cubic relationship with the center-to-center distance to the pickup coil in the long-distance limit. The reasoning behind this is as follows. Each of the 25 small 0.2 mm Amperian loops produces a dipole magnetic field. However, we can apply Stoke’s theorem to combine all the small loops into one big “effective” loop of 1 mm diameter. Essentially, the “internal” currents on the Amperian loops cancel out, leaving just an outer current. This single large effective magnetic dipole field should have an asymptotic inverse cubic decay in the long-distance limit, i.e., after a few millimeters. In the multipole series expansion of the magnetic vector potential of a general current loop, the inverse square component of the magnetic field is zero due to the absence of magnetic monopoles [29]. The inverse cubic (pure dipole), inverse quartic (pure quadrupole), and higher-order terms are generally non-zero for a finite loop and a finite current. The higher-order terms decay faster in the long-distance limit, leaving the inverse cubic term as the dominant term. That is, the amplitude of the total magnetic field from the NPs coating the clot is the approximate center-to-center distance $\overset{⃑}{r}$ dependence to the pickup coil

(7) $$B_{thrombus}\left(\overset{⃑}{r}\right)≃\frac{\alpha }{{\left(\beta +r\right)}^{3}}\;$$

where α is some normalization constant and β is an offset parameter in case the thrombus-center-to-center distance is different from the distance from the center of the coordinate system. Finally, we compute the total flux Φ through the pickup coil using the sum of dot products in Equation (6). The flux, which is essentially an average of the field over a small area, also has an asymptotic inverse cubic decay in the long-distance limit.

### 2.4. Three Orthogonal Pairs of Drive Coils for Thrombus Triangulation

Our primary goal is to monitor the distance between the thrombus and the stent to detect thrombus slippage or fragmentation (Figure 2). The challenge is that, due to possible bends in the artery, the orientation of the pickup coil relative to the thrombus could vary. Signal changes due to bends can confound thrombus slippage and fragmentation, so we need a methodology that can recover the distance and is robust to mild or even sharp bends in the artery. This can be achieved by using a set of three pairs of orthogonally oriented drive coils arranged outside the subject’s head, as shown in Figure 1. For the best results, these six external coils should be centered at the approximate location of the clot, but the method is robust and does not require high accuracy in placement or orientation.

### 2.5. Forward and Inverse Problems

**Figure 4:**
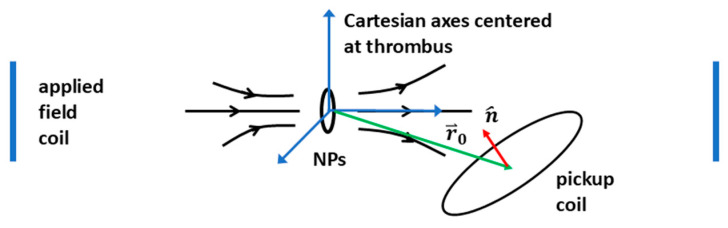
Schematic of geometry of NPs, the induced field, and pickup coil probe (not to scale). Only one pair out of the three pairs of applied field coils in the full setup is shown.

It is convenient to use Cartesian coordinates centered at and oriented with the thrombus for the calculations (Figure 4). The forward problem involves calculating the flux through the pickup coil of diameter 1 mm. The relevant variables are the distance vector ${\overset{⃑}{r}}_{0}$ of the center of the pickup coil relative to the center of the thrombus, and the unit vector $\hat{n}$ perpendicular to the pickup coil. These determine the flux through the pickup coil.

The forward problem is to calculate the flux Φ, given distance vector ${\overset{⃑}{r}}_{0}$ and orientation unit vector $\hat{n}$. The inverse problem is to recover ${\overset{⃑}{r}}_{0}$, given one or more measurements of Φ and $\hat{n}$. Since ${\overset{⃑}{r}}_{0}$ has three degrees of freedom in general, we need three independent flux measurements as mentioned before. The inverse problem can be solved using a “non-linear inversion” approach. The approach can be summarized as solving the forward problem for a grid of input values and finding the input value for which the output value is closest to the experimentally measured value.

### 2.6. Distance Recovery Method #1: Sum of 3 Orthogonal Fluxes

We first present a distance-recovery method that uses the three orthogonal-excitation fluxes only, without needing a measurement of $\hat{n}$. The advantage of this method is that it is simple and elegant. We calculate the sum of the absolute values of the three flux values from orthogonal excitations:

(8) $$\Phi_{3}=|\varphi_{X}|+|\varphi_{Y}|+\left|\varphi_{Z}\right|$$

For each orthogonal excitation $\varphi_{i}$, we use a combination of AC and DC fields of about 10 mT and 1 mT amplitudes, respectively. In previous studies, we have found that the combined usage of AC and DC driving fields is effective at extracting NP signal. The AC magnetic field frequency used was on the of order 1 kHz. These values have been found to work well for the 120 nm hydrodynamic diameter Micromod NPs that our lab has used over 10 years, leading to many publications. These NPs have a strong Brownian signal and negligible Neel signal. However, our methodology is not reliant on the size of the particle and should also work for other types of NPs, such as smaller particles with mostly Neel signal.

Suppose the AC field is oriented in the i-direction, the DC field can be in either of the two possible perpendicular directions. We have theoretical grounds, namely spatial symmetry, to expect $\Phi_{3}$ to be invariant with respect to changes in the pickup coil orientation when the pickup coil is sufficiently far away from the thrombus. We will quantify the validity of this premise in Section 3. This approximate invariance enables us to maintain a simple, one-to-one correspondence between $\Phi_{3}$ and thrombus–stent distance, within some small quantifiable error.

### 2.7. Distance Recovery Method #2: Rotation Correction

The second method to recover thrombus–stent distance from flux measurements is a refinement over the first method above. The additional step comprises turning down the pre-amplifier gain, disconnecting the cancelation coil, and measuring the large background signal due to the driving AC field (NP signal is negligible in this case). Experimentally, these steps can be automated and performed near instantaneously (e.g., every 1 s) because of our moderately fast field frequencies of around 1 kHz. Three of these measurements from orthogonal driving fields directly yield the unit vector $\hat{n}$ to high precision perpendicular to the pickup coil, within lab coordinates set by the directions of the three pairs of drive coils.

In addition, we measure $\varphi_{X},\;\varphi_{Y}$, and $\varphi_{Z}$ as before. As the stent is pulled, suppose a moment later the pickup orientation changes to a different vector ${\hat{n}}^{\prime }$ (e.g., due to a bend in the artery) and new fluxes are measured as well: ${\varphi^{\prime }}_{X},\;{\varphi^{\prime }}_{Y}$, and ${\varphi^{\prime }}_{Z}$. The inverse problem, i.e., finding the change in distance, can be solved using all these values with the non-linear inversion approach described above. The advantage of this method over the previous one is its theoretical infinite precision, since any rotation is explicitly corrected for. It would be limited only by experimental sources of error.

In this paper, we will begin with Method 1 and segue into and finish up with Method 2.

### 2.8. Experimental Setup

**Figure 5:**
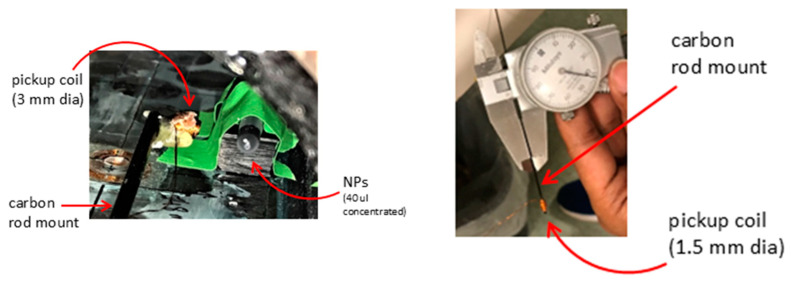
Photographs of the apparatus. (**Left**) A 3 mm diameter hand-wound pickup coil glued to a thin carbon fiber rod probe (black) and NP solution (black) inside a standard test tube. The setup exists inside the applied field coils and is mounted on a 2D translation stage (not shown). (**Right**) A 1.5 mm diameter pickup coil hand-wound on a 1 mm carbon fiber rod with a 36-gauge AWG fine copper wire (diameter 0.1 mm).

We used 40 μL of concentrated 25 mg/mL Micromod 100 nm diameter BNF-Dextran NPs without dilution in a 200 μL test tube to make as small and compact a magnetic field source as possible to emulate thrombus NP coating. Figure 5 shows a 3 mm and a 1.5 mm diameter hand-wound copper pickup coil mounted (glued with epoxy) to a long carbon fiber probe that is attached to a 2D translation stage. The NPs in the water sample are in a standard test tube. This setup exists almost at the same scale as the 1 mm diameter pickup coil required for a brain artery.

The pickup coil also receives a large background voltage from the external fields. We cancel out most if not all of the background signal using tunable cancelation coil(s) connected in series to the pickup coil. A pre-amplifier is used to boost the remaining signal. Finally, the lock-in amplifier digitizer isolates the NP signal from any remaining background signal. This methodology has been established in vitro; the experimental data are presented in later sections.

## 3. Results

### 3.1. NP Magnetization

**Figure 6:**
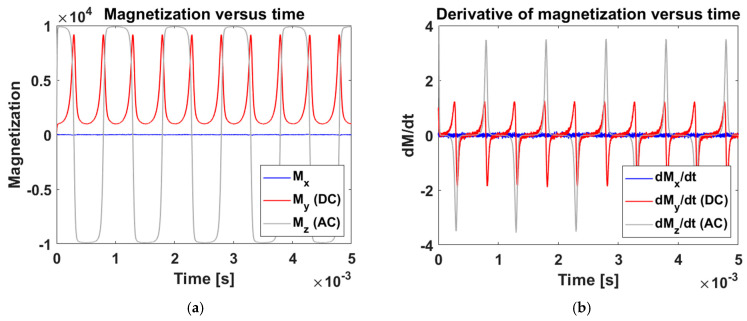
Simulation of the NP magnetization induced by a combination of 10 mT, 1 kHz sinusoidal AC field (z-direction), and a 1 mT DC field (y-direction), oriented perpendicular to each other. The voltage produced in the pickup coil resulting from the magnetization shown in panel (**a**) is shown in panel (**b**). For thrombus-sensing applications, we only care about the amplitude of the voltage, not the shape of the signal.

We acquired the signal from NPs in an alternating magnetic field. Figure 6 shows the NP magnetization as a function of time, simulated using a model for larger NPs where Brownian relaxation dominates. The signal settles down from a period of brief initial transient behavior into a regular periodic function. The waveform can be thought of as resulting from a balance between magnetic forces driving the NP rotation and frictional forces from the media the NPs are located in, which is generally aqueous solutions such as water or blood. The NPs try to follow the applied field so, at high frequencies, the magnetization approximates the sinusoidal applied field. At low frequencies and high fields, the NPs align with the applied field; when all the NPs are aligned, the magnetization reaches its maximum and is said to be saturated. The corners marking the transition of the region where the NPs are aligned with the increasing applied field and the region where all the NPs are aligned producing a flat magnetization produce higher harmonics, which are isolated from the current produced by the applied field in the pickup coils. This allows for very high sensitivity, similar to that achieved in MPI. The higher harmonics drop with increased relaxation times [30], which has been employed in magnetic spectroscopy of Brownian motion to measure media viscosity, temperature, and pH, as well biomarker concentrations via antibody-mediated aggregation [31].

The measured voltages in the pickup coil are proportional to the time derivatives of the magnetization (Equation (4)). The numerical derivative of the simulated magnetization in Figure 6a over the

(9) $$\frac{dM_{i}}{dt}\left(\frac{t_{n}+t_{n-1}}{2}\right)≃\frac{{M_{i}(t}_{n})-M_{i}(t_{n-1})}{\delta t}$$

small time increment $\delta t\equiv t_{n}-t_{n-1}$ accurately estimates the voltage. The results are shown in Figure 6b. Our numerical results for $M_{i}(t)$ have good stability; we estimate a 0.33% mean change in $\frac{dM_{i}}{dt}$ if δt is halved. In our simulation below, we use the maximum amplitude of $\frac{dM_{i}}{dt}$ in the third cycle when the signal has clearly stabilized from a period of brief initial transient behavior.

### 3.2. Simulation: Sum of Fluxes Versus Distance

**Figure 7:**
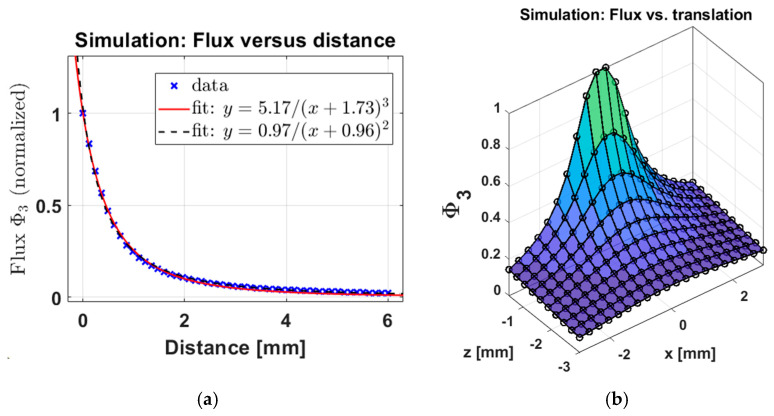
Simulated sum of fluxes, Φ_3, (**a**) as a function of one-dimensional translation distance, and (**b**) two-dimensional translation. In panel (**a**), both an inverse quadratic function with an offset and an inverse cubic function with an offset are fitted to the data. Both fits for the data are decent approximations but neither is perfect. This is expected from the multipole series expansion of a magnetic dipole field as a function of distance, which lacks a closed-form solution.

Figure 7a shows the simulated $\Phi_{3}$ (normalized to unity by dividing by the maximum value) versus distance for the hemispherical distribution of NPs shown in Figure 3. The distance corresponds to translation of the pickup coil from the z = 0 plane in Figure 3 towards the negative *z*-axis direction. We see that the inverse cubic function with an offset has a good fit to the data. We found that an inverse quadratic fit also fits the data well. This gives us a good basis for the relationship between distance and $\Phi_{3}$, which we will later compare with experimental data below.

It is important to understand how the flux varies as the pickup coil is moved away from the clot in longitudinal and transverse directions, and combinations thereof. Figure 7b shows flux as a function of longitudinal (*z*-axis) and transverse (*x*-axis) translations, with the pickup orientation fixed towards the *z*-axis. We see a similar drop-off rate in $\Phi_{3}$ in any translation direction. This demonstrates the direction-agnostic quality of our probe $\Phi_{3}$, which only considers the absolute distance between the center of the pickup coil and the center of the thrombus. We show the corresponding results with experimental data.

### 3.3. Quantifying Error in Distance Estimation from the Simulated Sum of Fluxes

**Figure 8:**
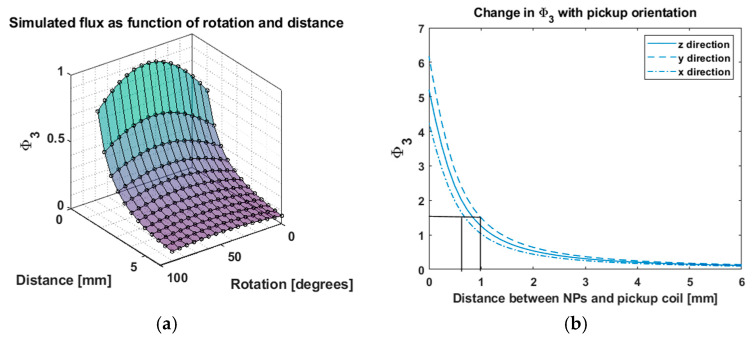
Simulated error in distance recovery with large changes in pickup orientation. In (**a**), we see $\Phi_{3}$ as a function of both distance between thrombus and pickup, and pickup coil orientation angle. We note that changing the orientation angle does not change the $\Phi_{3}$ as dramatically as does the distance. In (**b**), we estimate the change in $\Phi_{3}$ as the coil orientation is changed by 90 degrees about either axis. We find these large rotations corresponds to about a ±180 micron variation (black lines) in distance. This is our upper-bound estimate for distance recovery error using the sum of 3 fluxes, demonstrating its suitability for orientation-independent distance estimation.

We took a more comprehensive view by plotting flux as a function of both distance and rotation (Figure 8a). We found that the variation in $\Phi_{3}$ increases steadily with angle from zero degrees, reaching a maximum at 45 degrees and then decreasing to a value that is not quite the zero-degree value.

We quantified how robust $\Phi_{3}$ is to variations in pickup coil orientation, which can occur due to bends in the artery. Figure 8b shows $\Phi_{3}$ versus movement along the *z*-axis for three orthogonal pickup orientations. We estimated the range in $\Phi_{3}$ and found the corresponding error in distance recovery (black solid lines). The black lines simply visualize how the variation in flux relates to error in the recovered distance. We found approximately ±0.18 mm (180 micron) error, which is well below sub-millimeter precision. This is quite robust given the sharp, ninety-degree angle variations.

**Figure 9:**
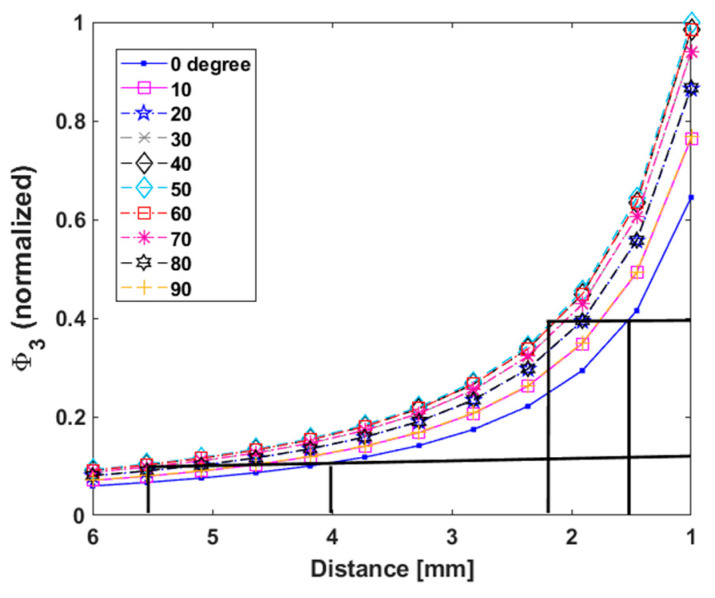
Simulated estimation of the precision of distance recovery from the sum of fluxes with variable pickup orientation angle (hemispherical distribution of NPs). The two sets of black lines demonstrate how the error in distance estimation increases for larger distances where the $\Phi_{3}$ has dropped significantly. This figure again highlights how the pickup coil orientation angle (shown in the legend) affects $\Phi_{3}$, but the impact of angle is much gentler than the impact of distance.

We analyzed these results further by plotting the same data as lines instead of a surface, as shown in Figure 9. We estimated the error in distance at close distances, e.g., around 1.5 mm, and found a maximum error of less than 1 mm (black lines). This error is small enough that $\Phi_{3}$ is relatively stable to pickup orientation changes, even if there are large, 45-degree bends in the artery. The stability is enough to reliably detect slippage, i.e., thrombus-SR detachment, or fragmentation. In other words, there is about a 40% maximum variation in $\Phi_{3}$ due to orientation changes, whereas there is approximately a 90% or higher drop in $\Phi_{3}$ if there is a 0.5 cm or higher distance between the pickup and clot, indicating slippage. Thus, these two effects, namely orientation change and slippage, should be easy to distinguish using $\Phi_{3}$ in a realistic situation.

The 40% number is a theoretical worst-case scenario. Generally, as long as the stent remains attached to the thrombus, the orientation of the pickup relative to the thrombus is expected to remain fairly steady as the stent is extracted from the artery, even through sharp arterial bends. To summarize, this analysis shows that monitoring $\Phi_{3}$ is suitable for detecting slippage, even without rotation correction, which we study next.

**Figure 10:**
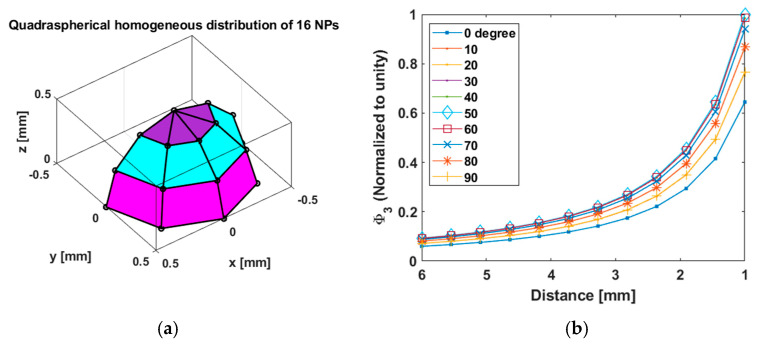
Simulated asymmetric, quadraspherical distribution of NPs, panel (**a**). Simulated flux versus distance when pickup orientation angle changes, panel (**b**). Upon comparison of (**b**) with the corresponding results for the hemispherical case (Figure 9), we find that the results are nearly identical. This suggests that the details of the distribution of NPs on the clot do not affect $\Phi_{3}$ significantly. This robustness is a desirable feature for our distance-sensing application.

These results remain true for asymmetrical NP coatings on the thrombus. In Figure 10, we show the results of a quadraspherical distribution. We found that they are nearly indistinguishable from those from the hemispherical case. The evidence gathered therefore suggests that $\Phi_{3}$ is a robust measure for slippage detection, since variations in the NP distribution on the clot are generally on a smaller scale (less than a millimeter) compared to the center-to-center distance (a few milimeters).

### 3.4. Simulated Rotation Correction

**Figure 11:**
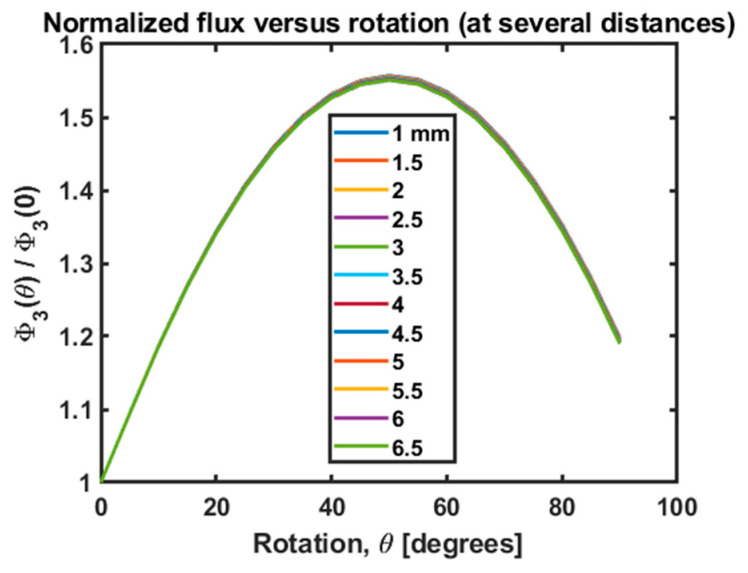
Simulated functional dependence of flux on rotation at multiple distances between the clot and the pickup coil. We see that if we normalize $\Phi_{3}$, the orientation dependence is identical at all distances. This is obvious from the separable nature of the two effects.

**Figure 12:**
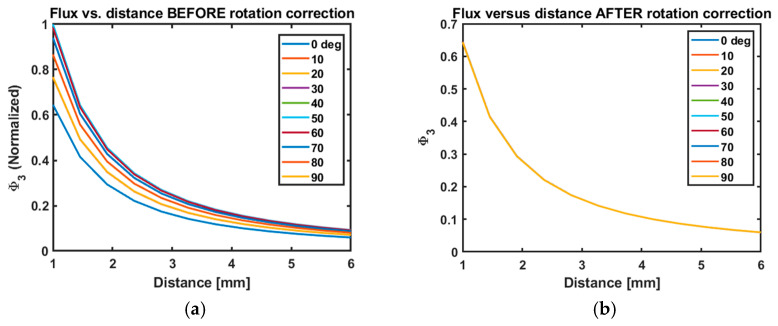
Simulated rotation correction. Since orientation and distance are separable, we can explicitly correct for orientation changes using the direct measurement of $\hat{n}$ discussed above. Panel (**b**) shows the result of the correction process on the data in (**a**).

Finally, the impact of changing the angle can be corrected in the sum of fluxes $\Phi_{3}$. Figure 11 shows $\Phi_{3}$ versus rotation for several distances. These data are the same as those presented in Figure 12, except they are normalized by $\Phi_{3}(\theta =0)$ to show the percentage change in $\Phi_{3}$. The curves are coincident, demonstrating that the rotation and distance dependence are separable, as expected.

Since the functional dependence of $\Phi_{3}$ on rotation is a one-to-one function for the domain below $45^{o}$, we utilize this function to adjust $\Phi_{3}$ for rotations in that domain. Pickup coil rotations can be deduced by successive measurements of the three background fluxes:

(10) $$\varphi_{bg:\;X}\;,\;\varphi_{bg:\;Y},\;\varphi_{bg:\;Z\;}⟶\;{\varphi^{\prime }}_{bg:\;X}\;,\;{\varphi^{\prime }}_{bg:\;Y},\;{\varphi^{\prime }}_{bg:\;Z\;}$$

The three background measurements give us the vector orientation of the pickup coil in the drive coil coordinate system, and thus the change in these gives us the change in the orientation of the pickup coil, e.g., as the stent moves through bends in the artery. Figure 12 shows the sum of fluxes $\Phi_{3}$ versus distance for different rotations before and after rotation correction. We note the perfect theoretical precision. This demonstrates the robustness of the rotation-corrected $\Phi_{3}$ methodology at recovering the distance between pickup coil and thrombus.

### 3.5. Experimental Results

**Figure 13:**
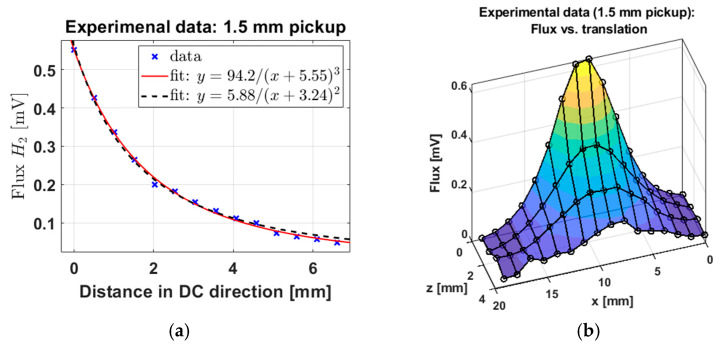

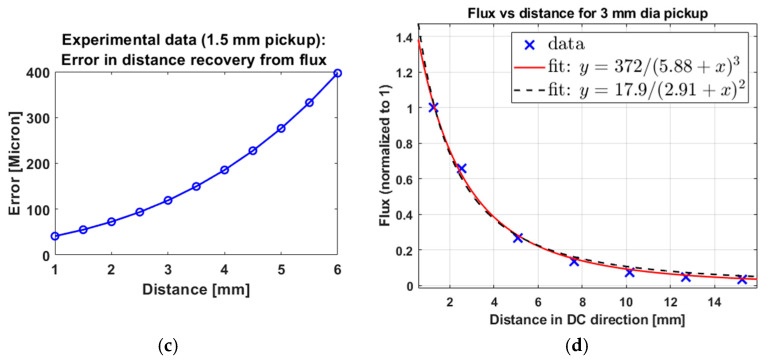
Experimental (in vitro) flux versus distance measurements for (**a**) 1.5 mm and (**d**) 3 mm diameter pickup coils. Panel (**b**) shows two-dimensional translations. Panel (**c**) shows the error in distance recovery as a function of the distance between the clot and the pickup coil. Panel (**a**) has an inverse quadratic and an inverse cubic fit, with computed $R^{2}$ values of 0.9965 and 0.9982, respectively, showing that the inverse cubic fit has a better fit. Panel (**d**) similarly shows the same two fits with $R^{2}$ values of 0.992 and 0.997, respectively, again showing that the inverse cubic fit agrees with the data slightly better. We note that visually, the inverse cubic fit is better in the long-distance limit compared to the inverse quadratic fit, consistent with the theoretical expectations for a magnetic dipole field.

Figure 13a,d shows the flux for a 1.5 mm and a 3 mm diameter pickup coil, respectively, as a function of distance. As with the simulated data above (Figure 7a), we see a good least-squares fit with the inverse cubic function with an x-intercept, discussed in Equation (7) above. The x-intercept was expected to be non-zero since the closest distance of the pickup coil to the center of the NP sample was non-zero (a few millimeters) due to the test tube wall in between (we measured distances starting from zero at this wall). The numerator is just a normalization constant. Both the pickup coil orientation and distance moved are in the DC field direction where the NP flux is maximum. The inverse cubic function was fitted using the default least-squares algorithm in MATLAB.

In Figure 13b, we show the full flux versus distance data for two-dimensional movement. The two-dimensional data have good consistency with the corresponding simulation data (Figure 7b). We see that the data have low noise near the NP sample and higher noise further out. This is consistent with the expected inverse cubic weakening of the signal with distance. Finally, in Figure 13c, we estimated the error in the distance recovery as follows. We calculated the RMS error of the data in Figure 13a after subtracting the inverse cubic fit. This RMS error in flux ${∆\Phi }_{3}$ was then translated into the RMS error in distance ∆D using the local slope, i.e., the derivative, of the fitted inverse cubic function. That is, for each distance D, we estimated the error as

(11) $$∆D={∆\Phi }_{3}/\left(\frac{d\Phi_{3}}{dD}\right)$$

The results are presented in Figure 13c. We calculated a distance error of 41 microns at 1 mm distance between the NPs on the clot and the pickup coil (i.e., probe). This value corresponds to a one-sigma error bar.

## 4. Discussion

The inverse cubic distance relationship means that if the flux through the probe drops by a factor of 8, we deduce that the center-to-center distance between the clot and pickup coil will increase by about a factor of 2. The relevant length scale is about 0.5 mm (Figure 3), which is roughly the closest realistic center-to-center distance between a 1 mm diameter clot and a 1 mm pickup, where the flux is maximum. If the thrombus becomes detached from the SR, we expect to see a dramatic drop in $\Phi_{3}$ as the distance increases from about 0.5 mm (stent touching the thrombus) to a few millimeters. We expect $\Phi_{3}$ to rapidly become a small fraction of its maximum value. This inherent high sensitivity to clot detachment is a strength of this technology. Essentially, we expect the flux to be high and maximum when the stent is attached to the clot, and nearly zero if they become detached.

The maximum value of $\Phi_{3}$ corresponds to the highest number of NPs and the closest distance between the pickup coil and clot. Fragmentation of the clot during extraction can be identified by the associated drop in pickup coil signal. Fragmentation and slippage both reduce the signal in the pickup coil, but fragmentation would probably be a more discrete event. In both cases, the force applied on the SR should probably be reduced to reduce slippage or fragmentation. It might be possible to move the SR back in an effort to reclaim the clot or reclaim the fragment if it has not moved too far. As the probe gets closer to the fragment or the slipped thrombus, the flux increases.

In the upcoming application phase, we will need to demonstrate that our targeted NPs bind successfully and efficiently to the clot in vivo. Antibody targeting of NPs involves some sophisticated chemistry. Our research group has made significant progress in antibody targeting [23]. In that work, NP antibody targeting was achieved with five different proteins in vitro, including Interleukin-6 (IL-6) which is a key inflammation biomarker molecule. The same technology should allow us to attach the NPs to the clot in vivo. This research is ongoing in our group.

NP safety is also an issue that needs to be addressed. In general, iron oxide NPs are attractive for human use [32] because they are generally non-toxic and biodegradable, so their use as an alternative to gadolinium in MRI [33] and their use in magnetic particle imaging [34] are being pursued. However, NP biocompatibility and safety depend on the surface chemistry in fairly specific ways [35], so specific antibody-targeted versions need to be evaluated for human use.

The targeted NPs would be single-use, i.e., used only once for each clinical patient. This is necessary to maintain a sterile surgical environment. We expect the targeted NPs to be reasonably priced (no more than a few hundred U.S. dollars, which was the cost for each synthesized batch in our preliminary, unpublished in vivo experiments), so that recycling to save costs is not necessary.

MT has significant advantages over the use of drugs to dissolve a clot. Our proposed technology could improve outcomes even further. Giving a physician feedback during the MT procedure allows the physician to adapt appropriately to unexpected events like very strong resistance to movement. The technology would keep the physician from proceeding blindly.

## 5. Conclusions

We simulated a hemispherical and a quadraspherical distribution of NPs coating the surface of a spherical thrombus and calculated the flux through a pickup coil as a function of distance to the thrombus and pickup coil orientation relative to external coils generating the applied field. Our goal was to guide the MT procedure, enabling a clinician to detect any slippage or fragmentation to allow them to compensate to the best extent possible. We found that the flux drops sharply as a function of the distance between the pickup coil and the thrombus approximately, with an inverse cubic fit as expected for a magnetic dipole. The high sensitivity is helpful in this application. We developed a general methodology to recover the center-to-center distance between a thrombus and the pickup coil by using three orthogonal flux measurements. We used simulations to show that our method is robust to mild and sharp bends in the brain artery. We showed via simulation that the distance can be recovered accurately with any arbitrary rotation of the pickup coil as it moves through tortuous arteries, with unlimited theoretical precision. We demonstrated through simulation and experimental data that the method is viable. This inexpensive magnetic NP technology for thrombus extraction assistance should improve treatment outcomes.

## Data Availability

Data are contained within the article.
